# Editorial: Physiological aspects of marathon running

**DOI:** 10.3389/fphys.2026.1790989

**Published:** 2026-02-09

**Authors:** Daichi Sumi, Yoshio Suzuki

**Affiliations:** 1 Institute of Sport Science, ASICS Corporation, Kobe, Japan; 2 Graduate School of Health and Sports Science, Juntendo University, Chiba, Japan

**Keywords:** endurance exercise, endurance performance, exercise physiology, gut, marathon

Marathon running represents an important domain within sports science, focusing on the physiological challenges and adaptive responses associated with sustained endurance activity. Current research themes include cardiovascular, neuromuscular, and metabolic processes engaged during prolonged running. Recent investigations have underscored the intricated interactions between energy pathways, fatigue development, and recovery mechanisms, in addition to highlighting the essential contributions of gastrointestinal function, nutrition, and hydration. In response, this Research Topic initiated to advance understanding of the physiological basis of marathon running through detailed exploration of the biological processes that underpin performance and adaptation.

The Research Topic contains six articles. The minireview by the two guest editors of this Research Topic provides an overview of the findings on gastrointestinal function and gut microbiota in relation to marathon running and highlights the unresolved issues requiring further research (Sumi and Suzuki). We hope that the perspectives presented in the minireview will form the basis for future research in this field. As a new finding related to probiotics, Cai et al. reported that *Lactobacillus pentosus* CQZC02 mitigates the decline in intestinal barrier function and exercise capacity caused by strenuous exercise in mice (Cai and Wang). Decrin secreted from myotubes in response to exercise stimuli plays a crucial role in the repair and regeneration of skeletal and cardiac muscle. De Fontes-Junior AA et al. demonstrated that blood levels of decorin in male marathon runners after a marathon race correlate with maximum speed, oxygen consumption, as well as parameters from cardiopulmonary exercise testing (CPET), such as VE/VCO_2_ and VE/VCO_2_ slope (de Fontes-Junior et al.). Hata et al. reported the development of the Hata-Yanagiya Physical Activity Calculation (HYPAC) system, which uses global positioning system (GPS) data to accurately estimate oxygen consumption during running and walking (Hata et al.). The HYPAC system could serve as an alternative to the ACSM’s conventional estimation formulas. The minireview by Grivas investigates the physiological process and psychological factors that underpin the effectiveness of negative split pacing in marathon running (Grivas). Evidence from futer competitive setting will be especially valuable in confirming that negative splits constitute a scientifically sound strategy for enhancing marathon. Finally, the work from Rapp et al. analyzed the determinants of running performance in cross-country events (Rapp et al.). The optimal performance prediction model included oxygen uptake at the estimated lactate threshold, the fat utilization rate during submaximal running, and running economy scaled allometrically. These six articles highlight the variety of perspectives on the physiology of the marathon running.

Taken together, these six articles highlight the range of perspectives necessary to advance our understanding of the physiology of marathon running. Sustained interest in this Research Topic, including manuscripts submitted beyond the initial deadline, resulted in several extensions and ultimately the launch Volume 2. Continued methodological development and scientific new view points are expected to further advance this field. Research Topic as an important venue fort highly quality research on the marathon running.

